# *In Vitro* Proliferation and Anti-Apoptosis of the Papain-Generated Casein and Soy Protein Hydrolysates towards Osteoblastic Cells (hFOB1.19)

**DOI:** 10.3390/ijms160613908

**Published:** 2015-06-17

**Authors:** Xiao-Wen Pan, Xin-Huai Zhao

**Affiliations:** 1 Key Laboratory of Dairy Science, Ministry of Education, Northeast Agricultural University, Harbin 150030, China; E-Mail: panxiaowen12358@126.com; 2 Synergetic Innovation Center of Food Safety and Nutrition, Northeast Agricultural University, Harbin 150030, China

**Keywords:** casein hydrolysates, soy protein hydrolysates, human fetal osteoblasts, proliferation, anti-apoptosis

## Abstract

Casein and soy protein were digested by papain to three degrees of hydrolysis (DH) 7.3%–13.3%, to obtain respective six casein and soy protein hydrolysates, aiming to clarify their *in vitro* proliferation and anti-apoptosis towards a human osteoblastic cell line (hFOB1.19 cells). Six casein and soy protein hydrolysates at five levels (0.01–0.2 mg/mL) mostly showed proliferation as positive 17β-estradiol did, because they conferred the osteoblasts with cell viability of 100%–114% and 104%–123%, respectively. The hydrolysates of higher DH values had stronger proliferation. Casein and soy protein hydrolysates of the highest DH values altered cell cycle progression, and enhanced cell proportion of S-phase from 50.5% to 56.5% and 60.5%. The two also antagonized etoposide- and NaF-induced osteoblast apoptosis. In apoptotic prevention, apoptotic cells were decreased from 31.6% to 22.6% and 15.6% (etoposide treatment), or from 19.5% to 17.7% and 12.4% (NaF treatment), respectively. In apoptotic reversal, soy protein hydrolysate decreased apoptotic cells from 13.3% to 11.7% (etoposide treatment), or from 14.5% to 11.0% (NaF treatment), but casein hydrolysate showed no reversal effect. It is concluded that the hydrolysates of two kinds had estradiol-like action on the osteoblasts, and soy protein hydrolysates had stronger proliferation and anti-apoptosis on the osteoblasts than casein hydrolysates.

## 1. Introduction

Osteoporosis as a systemic skeletal disease is characterized by low bone mass and micro-architectural deterioration of bone tissue, with a consequent increase in bone fragility and susceptibility to fracture [1]. Osteoporosis is one of these serious health problems and reported to affect millions of people all over the world. Bone growth is maintained by two coordinated actions of osteoblasts and osteoclasts. In the body, osteoblasts are responsible for bone formation while the duty of osteoclasts is bone resorption. Imbalance between the osteoblasts and osteoclasts leads to osteopetrosis or osteoporosis, by changing bone mass [2]. Traditional medication for osteoporotic treatment is divided into three parts: antiresorptive, bone-forming and mineralization drugs. However, some side effects, such as the risk of breast and endometrial cancers caused by the estrogen and the gastrointestinal discomfort caused by the bisphosphonates, are still plaguesome at the present time [3]. It is beneficial to develop functional food ingredients with bone health function but without any side effect.

Both food proteins and amino acids are essential materials for the organic synthesis in the bone. Lacking protein long-term can cause a decrease in bone matrix synthesis, leading to osteoporosis. For example, the animals with lower protein feeding (13% of total feed) for five months have shown an increased bone resorption [4]. On the contrary, high protein intake is also detrimental to bone health, as it causes greater calcium losing and bone absorption, together with an inhibition on matrix mineralization [5]. The role of food protein in bone health therefore needs a long-term systematic investigation and experimental evaluation.

Decomposed products of food protein with lower molecular weights possess biological activities, and are known as bioactive peptides. Bioactive peptides, mostly obtained by enzymatic hydrolysis or microbial fermentation of protein substrates, play important roles in metabolism and physiological regulation, and their bioactivities, such as antihypertension, antioxidation, immuno-modulation and others, have become the most popular topic in the field of food science. Bioactive peptides have the potential to become health ingredients and nutraceutical preparations [6]. Enzymatic hydrolysis of food proteins offers a rapid and reproducible approach to generate bioactive peptides. Some bioactive peptides from plant and animal protein origins have been found to have an effect on bone health. Casein phosphorpeptides are able to promote calcium absorption and utilization [7], while soy protein hydrolysates are capable of reducing bone turnover for the postmenopausal women [8]. In short, the effects of protein hydrolysates on bone health have been proven. However, a comparison of potential *in vitro* effects of protein hydrolysates on the osteoblast in view of cell proliferation, cycle progression and apoptotic antagonism (*i.e.*, apoptotic prevention and apoptotic reversal) are not well-clarified yet. A detailed investigation is therefore absolutely needed.

In the present study, casein and soy protein were digested *in vitro* by papain into three degrees of hydrolysis (DH). Six hydrolysates obtained were divided into two groups based on their origins, and evaluated for their cell proliferation on a human fetal osteoblastic cell line (hFOB1.19 cells) *in vitro*, aiming to select the hydrolysate of the strongest cell proliferation from each group. Moreover, two hydrolysates selected were evaluated for their *in vitro* effects on cell cycle distribution and apoptosis of the cells induced by two chemicals (etoposide and NaF, respectively). The aim of the present study was to reveal protein hydrolysates’ proliferation and anti-apoptosis on the osteoblasts as well as the potential effect on the bone health.

## 2. Results and Discussion

### 2.1. In Vitro Proliferation of the Six Hydrolysates towards hFOB1.19 Cells

**Figure 1:**
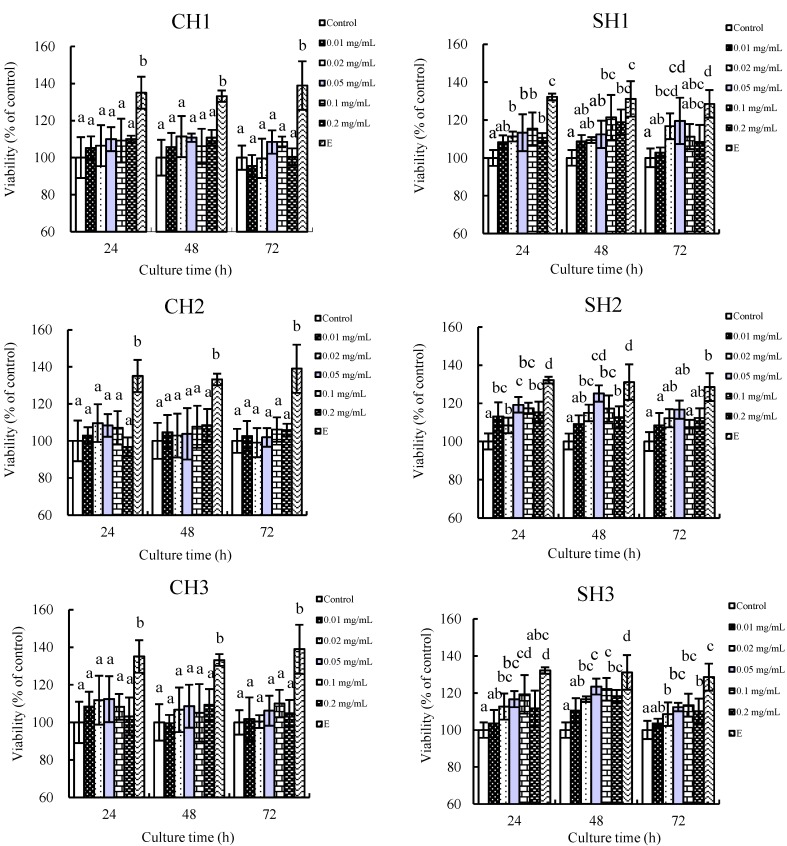
Proliferation of casein and soy protein hydrolysates (CH1–3 and SH1–3) on hFOB1.19 cells cultured for 24, 48 and 72 h, respectively. E denotes 17β-estradiol. CH1–3 denote casein hydrolysates with DH values of 7.3%, 10.8% and 13.3%, while SH1–3 denote soy protein hydrolysates with DH values of 7.5%, 10.9% and 13.2%, respectively. Different lowercase letters above the columns indicate that the mean values of different groups are significantly different (*p* < 0.05).

Six protein hydrolysates were prepared in the present study from casein and soy protein. The data given in Table 1 show their measured DH values (*i.e.*, hydrolysis extent). The six hydrolysates were added to the cell culture at 0.0–0.2 mg/mL to assay their *in vitro* proliferation towards the hFOB1.19 cells. After culture time of 24–72 h, cell viability of the osteoblasts in each group was assayed (shown in Figure 1). Almost all hydrolysates promote *in vitro* osteoblastic proliferation as the used positive chemical 17β-estradiol dose. The measured cell viability is 100%–114% and 104%–123% when casein and soy protein hydrolysates are added into the cultures. These data show that both casein and soy protein hydrolysates have estradiol-like action. However, soy hydrolysates (SH) totally show greater proliferation than casein hydrolysates (CH). More importantly, the hydrolysates with higher DH values seem to possess stronger osteoblast proliferation, that is, CH3 and SH3 show much more powerful proliferation than their counterparts with lower DH values (*p* < 0.05). At the same time, it can be seen that the six hydrolysates mostly exhibit greater osteoblast proliferation (*p* < 0.05) when hydrolysate addition and culture time are 0.05 mg/mL and 48 h, respectively. It is thus briefly concluded that the hydrolysates prepared in the present study could enhance osteoblast growth and protein origin; hydrolysis extent (*i.e.*, DH) and addition level are important to the measured proliferation. Two hydrolysates, CH3 and SH3, have the strongest proliferation compared to their counterpart hydrolysates of lower DH values; thus, they are selected as target hydrolysates in the forthcoming study.

**Table 1:** Hydrolysis conditions and degrees of hydrolysis (DH) of the prepared six hydrolysates.

Hydrolysates	Hydrolysis Times (h)	Other Conditions	DH (%)
CH1	1.5	pH 6.0, 60 °C, papain of 2 kU/g protein	7.3
CH2	3.5		10.8
CH3	7.0		13.3
SH1	3.0		7.5
SH2	5.0		10.9
SH3	7.0		13.2

CH and SH denote casein and soy protein hydrolysates, respectively.

Bone remodeling occurs continuously to maintain normal bone mass in the whole life period [9]. Osteoblasts are bone-forming cells, and osteoblast proliferation is absolutely an important stage in bone formation. In fact, casein and soy protein hydrolysates have been studied to show their beneficial effects on the bone. For example, the two kinds of protein hydrolysates can accelerate bone turnover with formation exceeding resorption and lead to overall gains in density [10,11], providing a support to the present result. Furthermore, in comparison with casein hydrolysates, soy protein hydrolysates tend to increase sIGF-1, a key regulator of bone metabolism [12,13]. This could partly explain the present CCK-8 assaying results that soy protein hydrolysates have greater ability to promote osteoblast proliferation. The protein hydrolysates investigated in the present study exert different effects on osteoblast proliferation, if they have similar DH values. This might be caused by the different amino acid composition of the hydrolysates, which should be investigated in later study. Why hydrolysis extent (*i.e.*, DH) of protein hydrolysates affects osteoblast proliferation remains to be an unexplored topic, and also should be clarified in future.

### 2.2. Effects of the Two Hydrolysates on Cell Cycle Distribution of hFOB1.19 Cells

**Figure 2:**
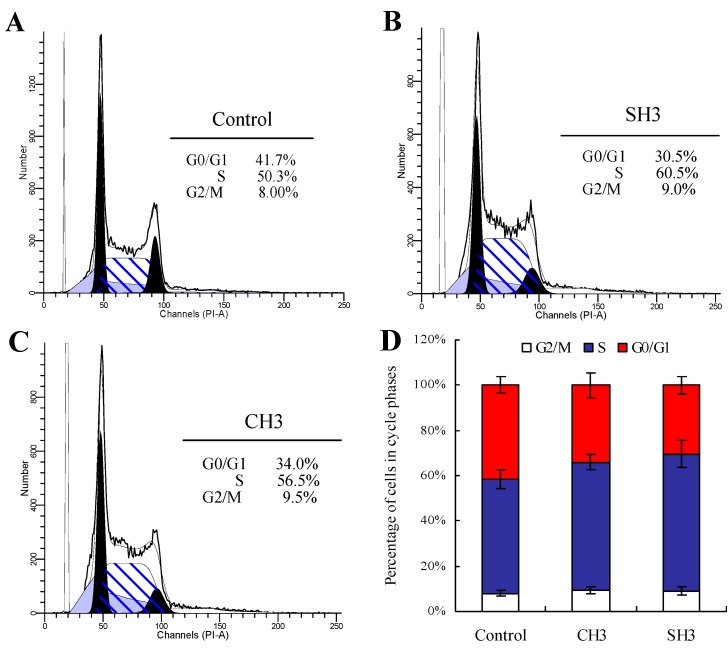
Effects of casein and soy protein and hydrolysates (CH3 and SH3) on cell cycle phase distribution of hFOB1.19 cells. (**A**) Representative histograms of DNA content in control group cells; (**B**,**C**) Representative histograms of DNA content in the cell incubated with CH3 and SH3 at 0.05 mg/mL for 48 h. Horizontal and vertical axes indicate the relative nuclear DNA content and number of cells, respectively; and (**D**) Percentage of cell populations in G0/G1-, S- and G2/M-phases.

The effects of CH3 and SH3 on cell cycle progression of the osteoblasts were monitored. Flow cytometry analysis results are given in Figure 2, which reveals the distribution of the cells in G0/G1-, S-, and G2/M-phases. Compared to the cell cycle distribution of the cells in control group, CH3 and SH3 have no effect on the ratio of G2/M-phase cells, but elevate the ratio of S-phase cells and decrease the ratio of G0/G1-phase cells. SH3 exhibits the more potent capacity to arrest more cells in S-phase (60.5%); however, CH3 shows the weaker one (56.5%). This result is consistent with the proliferation of the two hydrolysates measured before. It is thus concluded that CH3 and SH3 confer the hFOB1.19 cells with cell proliferation via an alteration of cell cycle in the S-phase.

Cell cycle is a well-ordered sequence of biochemical events, occurring in eukaryotic cells between one cell division and the next [14]. More cells are in S-phase (DNA synthesis), which suggests that more cells are ready for proliferation. Some studies have reported the effects of collagen and gelatin hydrolysates on cell cycle progression, stating that they can promote osteoblast proliferation by increasing the ratio of S-phase cells [15,16]. Similarly, a combination of 17β-estradiol and testosterone (two chemicals with osteoblast proliferation) also have been observed to enhance the ratio of S-phase cells effectively [17]. The present result is consistent with the past ones, clarifying an intrinsic effect of casein and soy protein hydrolysates on the hFOB1.19 cells by arresting cell cycle in S-phase.

### 2.3. Anti-Apoptosis of the Two Hydrolysates towards hFOB1.19 Cells

*In vitro* antagonism of CH3 and SH3 towards EP- and NaF-induced apoptosis of the osteoblasts was investigated by assaying the ratio of apoptotic cells, using Annexin V-FITC/PI double staining and flow cytometric analysis. Previous evaluation showed that EP and NaF at 10 and 40 mg/mL, respectively, were capable of inducing osteoblast apoptosis. The two addition levels were thereof applied to treat the cells in the present study. After being treated with 0.05 mg/mL CH3 or SH3 for 48 h, and then with EP or NaF for 24 h respectively, the cells were double stained and assayed to reveal apoptotic prevention of CH3 and SH3. Otherwise, after being first treated with EP or NaF for 24 h, and then with 0.05 mg/mL CH3 or SH3 for 48 h, respectively, the cells were also double stained and assayed to reveal apoptotic reversal of CH3 and SH3.

**Figure 3:**
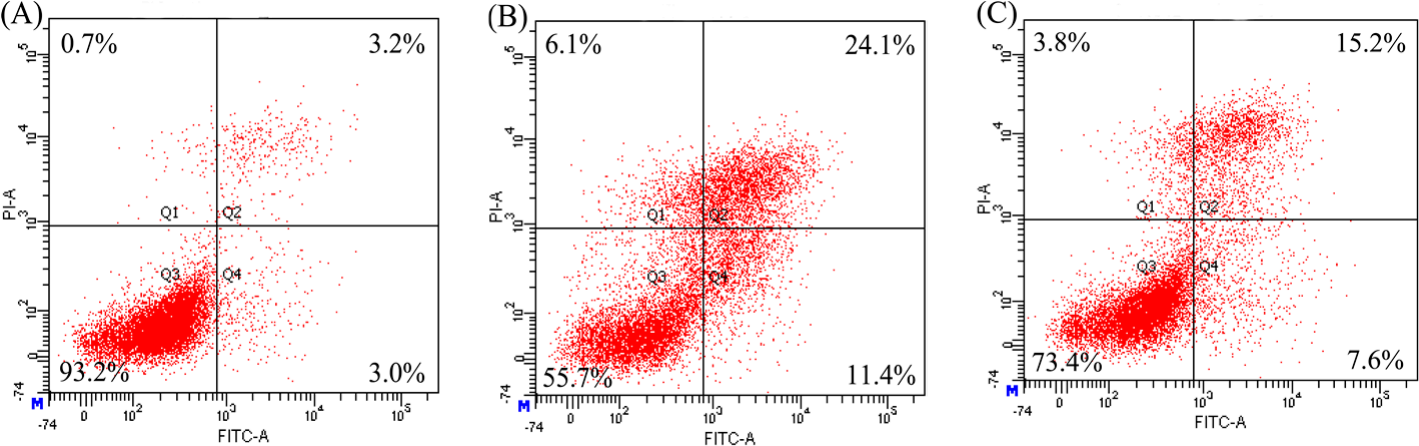

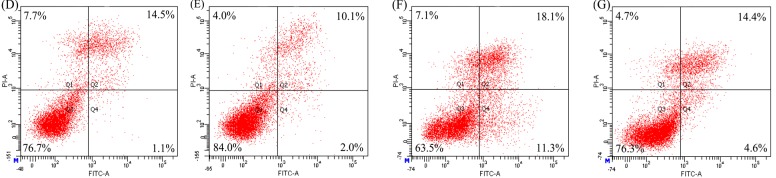
Apoptotic prevention of casein and soy protein hydrolysates (CH3 and SH3) at 0.05 mg/mL on etoposide- and NaF-induced apoptosis of hFOB1.19 cells. Cell apoptosis was measured by Annexin FITC-V/PI double-stained flow cytometry. Q1, dead cells; Q2, later apoptotic cells; Q3, viable cells; Q4, early apoptotic cells. (**A**) The cells in the control group; (**B**,**C**) The cells treated by etoposide and NaF, respectively; (**D**,**F**) The cells first treated by CH3 and SH3, respectively, and then by etoposide; and (**E**,**G**) The cells first treated by CH3 and SH3, respectively, and then by NaF. The labeled data were obtained from one assaying only.

**Figure 4:**
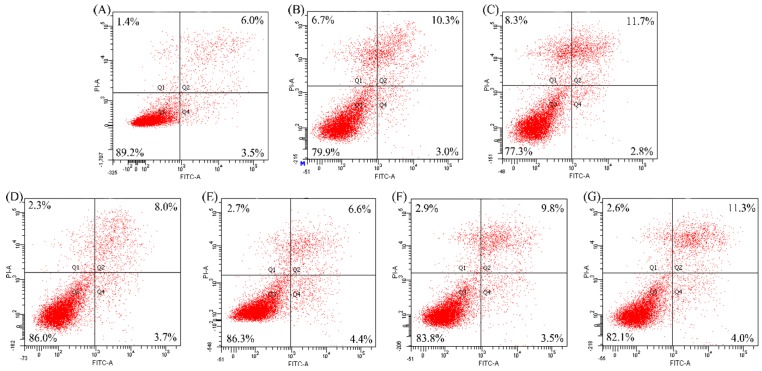
Apoptotic reversal of casein and soy protein hydrolysates (CH3 and SH3) at 0.05 mg/mL on etoposide- and NaF-induced apoptosis of hFOB1.19 cells. Cell apoptosis was measured by Annexin FITC-V/PI double-stained flow cytometry. Q1, dead cells; Q2, later apoptotic cells; Q3, viable cells; Q4, early apoptotic cells. (**A**) The cells in control group; (**B**,**C**) The cells treated by etoposide and NaF, respectively; (**D**,**F**) The cells first treated by etoposide, and then by CH3 and SH3, respectively; and (**E**,**G**) The cells first treated by NaF, and then by CH3 and SH3, respectively. The labeled data were obtained from one assaying only.

The results given in Figure 3 show that both CH3 and SH3 have ability to decrease apoptotic cells, that is, to provide apoptotic prevention for the osteoblasts towards EP- and NaF-induced apoptosis. Based on these assaying results, a statistical analysis is carried out (as shown in Table 2). It is seen that application of CH3 and SH3 results in the ratios of apoptotic cells (Q2 + Q4) decreased from 31.6% (EP model group) and 19.5% (NaF model group) into 22.6% and 15.1% (EP treatment), or into 17.7% and 12.4% (NaF treatment), respectively. SH3 is more potent than CH3, as much less apoptotic cells are detected during assaying. The results given in Figure 4 show that SH3 other than CH3 has the ability to decrease apoptotic cells; that is, only SH3 has a reversal effect on apoptotic osteoblasts. It is seen from the data that application of SH3 results in the ratios of apoptotic cells decreased from 13.3% (EP model group) and 14.5% (NaF model group) into 11.7% (EP treatment) and 11.0% (NaF treatment), while application of CH3 results in the ratios of apoptotic cells about 13.3% (EP treatment) and 15.3% (NaF treatment) (Table 2). Overall, it is seen that SH3 exhibits more powerful protection and reversal effects on the two induced apoptosis than CH3, conferring the treated osteoblasts with less apoptotic cells in any case.

**Table 2:** Anti-apoptosis of casein and soy protein hydrolysates (CH3 and SH3) towards etoposide (EP)- or NaF-induced apoptosis of hFOB1.19 cells.

Modes	Apoptosis Inducers	Percentage of Apoptotic Cells (%)		
		Model Group	CH3 Group	SH3 Group
Apoptotic prevention	EP	31.6	22.6	15.1
	NaF	19.5	17.7	12.4
Apoptotic reversal	EP	13.3	13.3	11.7
	NaF	14.5	15.3	11.0

CH3 and SH3 are casein and soy protein hydrolysates with DH of 13.3% and 13.2%, respectively. Apoptotic cells are the total of later (Q2) and early (Q4) apoptotic cells shown in Figure 3 and Figure 4.

Apoptosis is a morphologically identifiable form of programmed cell death in the mammals [18]. It is recognized now that many factors regulate the apoptosis of bone cells [19]. The prevention and reversal for osteoblast apoptosis are expected importantly to prevent osteoporosis. NaF can induce osteoblast apoptosis [20]. EP is an anti-neoplastic agent and epipodophyllotoxin used in cancer treatment, also has dose-related inhibition on osteoblasts [21]. EP and NaF thus are capable of inducing osteoblast apoptosis, evidenced again by the present study. Fortunately, some proteins such as lactoferrin and thrombin were found able to inhibit osteoblast apoptosis [22,23], which gave the same conclusion as the present study. Antioxidation, antihypertension and immuno-modulation of protein hydrolysates had been widely studied in the past [24,25,26]. However, less attention has been paid to anti-apoptosis of protein hydrolysates towards osteoblasts and other cells. The finding of anti-apoptosis of casein and soy protein hydrolysates towards osteoblasts might initiate a novel perspective of food research. Much work should be carried out for other hydrolysates and cells, especially, why soy protein hydrolysates had a stronger effect than casein hydrolysates is still a scientific mystery.

## 3. Materials and Methods

### 3.1. Materials and Reagents

Casein was obtained from Beijing Aoboxing Biotechnologies Inc. (Beijing, China). Soy protein was prepared from defatted soy flour with a procedure described by Jiang and Zhao [27]. Fetal bovine serum (FBS) was purchased from Thermo Fisher Scientific Inc. (Cleveland, OH, USA). Papain was obtained from Sinopharm Chemical Reagent Co., Ltd. (Shanghai, China). Dextran T-70, activated charcoal, G418, etoposide (EP) and DMEM:Ham’s F12 (1:1) medium were obtained from Sigma Chemical Co. (St. Louis, MO, USA). Cell Counting Kit-8 (CCK-8) and Annexin V-FITC/PI Kit were purchased from Dojindo Molecular Technologies, Inc. (Kyushu, Japan) and 4 A Biotech Co., Ltd. (Beijing, China), respectively. The following reagents were purchased from Solarbio Science and Technology Co., Ltd. (Beijing, China): trypsin-EDTA, propidium iodide (PI), RNase A reagent. The water used was redistilled or ultrapure water, while other chemicals used were analytical grade.

Charcoal/dextran treated FBS (CD-FBS) was performed using a modified method of Eckert and Katzenellenbogen [28]. In brief, charcoal (250 mg) and dextran (25 mg) were mixed with the FBS (100 mL) and incubated at 55 °C for 45 min; after that, centrifugal separation of the FBS and insolubles were carried out at 9000× *g* for 15 min. The above steps were repeated three times to get sterilized serum by filtration through a 0.22 μm filter.

Human fetal osteoblast cell line (hFOB1.19 cells) was obtained from Cell Bank of the Chinese Academy of Sciences (Shanghai, China). The cells were derived from primary cultures of the tissue of fetal limbs and conditionally immortalized with a gene coding for a temperature-sensitive mutant (tsA58) of the SV40 large T-antigen along with a gene coding for neomycin (G418) resistance.

### 3.2. Preparation of the Hydrolysates

Casein and soy protein of 5 g (on dry basis) were dissolved in 100 mL water, respectively. The solutions were adjusted to pH 6.0 by adding 1 mol/L NaOH or HCl, kept at water bath with a set temperature of 60 °C, and then added by papain of 2 kU/g protein. The used papain is specified with preferential cleavage on the peptide bonds formed by Arg, Lys, and Phe-X [29]. After hydrolysis times of 1, 2, 3, 4, 5, 6 and 7 h, hydrolyzed protein solutions of 15 mL were separated and heated at boiling water bath for 15 min to terminate the hydrolysis. After cooling to room temperature, hydrolyzed protein solutions were centrifuged at 11,000× *g* for 20 min. The supernatants (protein hydrolysates) collected were evaluated for protein content and free amino groups to calculate DH values. Based on the assaying results, each protein was hydrolyzed at the same conditions but with different times, to generate three hydrolysates of different DH values. Six hydrolysates thus prepared were lyophilized, dried and stored at −20 °C before use.

### 3.3. Chemical Analyses

Nitrogen content was assessed using the Kjeldahl method in the Kjeltec 2300 Protein Analyzer (Foss Tecator AB, Hoganas, Sweden), and multiplied by conversion factors of 6.38 and 5.71 to obtain respective content of casein and soy protein, respectively [30].

Free amino groups were assayed by the *o*-pthaldialdehyde (OPA) method [31] with slight modifications. The assaying was carried out by adding hydrolysate (or standard) solution of 3 mL to the OPA reagent of 3 mL. Absorbance of the mixed solution was measured at 340 nm by a UV spectrophotometer (UV-2401PC, Shimadzu, Tokyo, Japan) after a reaction time of 5 min. l-Leucine solutions of 6–30 μg/mL were used as standards. DH value was calculated as DH (%) = (*h*/*h*_tot_) × 100. The suggested *h*_tot_ values were 8.2 and 7.8 meq/g protein for casein and soy protein [32], respectively.

### 3.4. Cell Culture Conditions and Cell Viability Assaying

The cells were cultured in DMEM: Ham’s F-12 (1:1) medium without phenol red supplemented with 10% (*v*/*v*) CD-FBS and 0.3 mg/mL G418, and maintained at 34 °C in a humidified atmosphere containing 5% CO_2_.

Cell viability was assayed by a CCK-8 method. CCK-8 contains WST-8 reagent that can be reduced into a kind of yellow water-soluble compounds (formazan) by dehydrogenase in the mitochondria [33]. Amount of the generated formazan is proportional to the number of living cells. The cells were seeded at 5 × 10^3^ cells per well in 96-well plates and incubated at 34 °C in normal culture medium. After 24 h, the cells were starved with DMEM:Ham’s F-12 medium (1:1) containing 0.5% CD-FBS overnight. After then, the cells were treated with each hydrolysate of six levels (0, 0.01, 0.02, 0.05, 0.1 and 0.2 mg/mL) and 17β-estradiol (E) (10^−8^ mol/L) for 24–72 h. As a positive control, E was dissolved in DMSO and made up with the medium so that final concentration of the vehicle was less than 0.1%. After the incubation, 20 μL CCK-8 was added to each well and incubated for another 4 h; afterwards, the absorbance was measured by a microplate reader (Bio Rad Laboratories, Hercules, CA, USA) at a wavelength of 450 nm. The cells without hydrolysates or E treatment were as 100% viable.

### 3.5. Cell Cycle and Apoptosis Assaying

Cell cycle distribution was detected by the classical propidium iodide (PI) staining method and flow cytometric analysis. The cells were seeded in 6-well flat-bottomed plates at 3 × 10^5^ cells per well for 24 h in normal culture medium, and then another 24 h in serum-deprived medium to synchronize cells at G0-phase. After that, the cells were exposed to the selected hydrolysates at 0.05 mg/mL for 48 h. The cells without hydrolysate treatment were served as the control. All cells were harvested by trypsinization, washed with phosphate-buffered saline (PBS) twice, suspended in chilled 70% ethanol at 4 °C overnight, and then re-suspended in the solution containing 50 μg/mL PI, Triton X-100 (0.1%) and RNase A (20 μg/mL) for 30 min at 37 °C in a dark room. For each experiment, 3000 events per sample were recorded by the flow cytometry (FACS Calibur, Becton Dickson, San Jose, CA, USA). Proportion of the cells in G0/G1-, S-, and G2/M-phases were analyzed by the ModFit software (Verity Software House, Topsham, ME, USA).

Two fluorescent dyes, Annexin V-FITC and PI, were used to detect the apoptotic and necrotic cells. Annexin V-FITC identifies early and late apoptotic cells, while late apoptosis and necrotic cells are stained by PI. The protocol used in the present analysis was recommended by the kit manufacturer. Briefly, the experiment was divided into two groups: apoptotic prevention group and apoptotic reversal group. In apoptotic prevention group, the cells were grown to about 80% confluence in the 6-well plates and treated with the hydrolysates for 48 h, followed by 24 h treatment with the proapoptotic agent EP (10 mg/L) and NaF (40 mg/L), respectively. While in apoptotic reversal group, two proapoptotic agents were added before hydrolysates. Those cells without any treatment served as the control group. After treatment, the cells in each group were harvested by trypsinisation, washed twice with PBS and re-suspended in 200 μL binding buffer containing 10 μL Annexin V-FITC and 5 μL PI. The samples were incubated in the dark at room temperature for 15 min, and then analyzed on the flow cytometry. The number of intact cells, early and late apoptotic cells and necrotic cells were discriminated by counting the cells directly.

### 3.6. Statistical Analyses

All data are expressed as means or means ± standard derivations (SD) from three independent experiments and analyses, and statistical significance between different groups was analyzed by one-way analysis of variance (ANOVA) with Duncan’s multiple range tests. Statistical significance was defined as a *p* < 0.05, with Duncan procedure of the SPSS version 13.0 program (SPSS Inc., Chicago, IL, USA).

## 4. Conclusions

The papain-generated casein and soy protein hydrolysates have estradiol-like action *in vitro*, and could promote the growth of human fetal osteoblasts (hFOB1.19 cells). The assessed proliferation depends on protein origins, hydrolysis extent and addition levels of the hydrolysates. Two hydrolysates of the highest DH values can arrest cell cycle progression in S-phase to favor cell proliferation, and antagonize NaF- and etoposide-induced apoptosis shown as apoptotic prevention or reversal for the osteoblasts. Soy protein hydrolysates totally show powerful proliferation and anti-apoptosis compared to casein hydrolysates. In total, the present results provide scientific evidence to show new bio-function of protein hydrolysates; that is, they are beneficial to the bone via proliferation and anti-apoptosis towards the osteoblasts.

## References

[1] Adamson N.J., Reynolds E.C. (1996). Characterization of casein phosphopeptides prepared using alcalase: Determination of enzyme specificity. Enzym. Microb. Technol..

[2] Henderson J.E., Kremer R., Goltzman D., Bilezikian J.P., Raisz L.G., Rodan G.A. (2002). Systemic factors in skeletal manifestations of malignancy. Principles of Bone Biology.

[3] Reid I.R. (2013). Osteoporosis treatment: Focus on safety. Eur. J. Int. Med..

[4] Mardon J., Habauzit V., Trzeciakiewicz A., Davicco M.J., Lebecque P., Mercier S., Tressol J.C., Horcajada M.N., Demigné C., Coxam V. (2008). Influence of high and low protein intakes on age-related bone loss in rats submitted to adequate or restricted energy conditions. Calcif. Tissue Int..

[5] Krieger N.S., Frick K.K., Bushinsky D.A. (2014). Mechanism of acid-induced bone resorption. Curr. Opin. Nephrol. Hypertens..

[6] Hartmann R., Meisel H. (2007). Food-derived peptides with biological activity: From research to food applications. Curr. Opin. Biotechnol..

[7] Donida B.M., Mrak E., Gravaghi C., Villa I., Cosentino S., Zacchi E., Perego S., Rubinacci A., Fiorilli A., Tettamanti G. (2009). Casein phosphopeptides promote calcium uptake and modulate the differentiation pathway in human primary osteoblast-like cells. Peptides.

[8] Evans E.M., Racette S.B., van Pelt R.E., Peterson L.R., Villareal D.T. (2007). Effects of soy protein isolate and moderate exercise on bone turnover and bone mineral density in postmenopausal women. Menopause.

[9] Zhang J.C., Liu C.L., Li Y.P., Sun J., Wang P., Di K.Q., Chen H., Zhao Y.Y. (2010). Effect of cerium ion on the proliferation, differentiation and mineralization function of primary mouse osteoblasts *in vitro*. J. Rare Earths.

[10] Arjmanoi B.H., Alekel L., Hollis B.W., Amin D., Stacewicz-Sapuntzakis M., Guo P., Kukreja S.C. (1996). Dietary soybean protein prevents bone loss in an ovariectomised rat model of osteoporosis. J. Nutr..

[11] Budek A.Z., Bjornvad C.R., Molgaard C., Bügel S., Vestergaard M., Pulkkinend P., Michaelsen K.F., Sangild P.T. (2007). Effects of casein, whey and soy proteins on volumetric bone density and bone strength in immunocompromised piglets. e-SPEN Eur. J. Clin. Nutr. Metab..

[12] Canalis E., Pash J., Varghese J.S. (1993). Skeletal growth factors. Crit. Rev. Eukaryot. Gene Expr..

[13] Rizzoli R., Bonjour J.P. (2004). Dietary protein and bone health. J. Bone Miner. Res..

[14] Kelly G.M., Kilpatrick J.I., van Es M.H., Weafer P.P., Prendergast P.J., Jarvis S.P. (2011). Bone cell elasticity and morphology changes during the cell cycle. J. Biomech..

[15] Tsuruoka N., Yamato R., Sakai Y., Yoshitake Y., Yonekura H. (2007). Promotion by collagen tripeptide of type I collagen gene expression in human osteoblastic cells and fracture healing of rat femur. Biosci. Biotechnol. Biochem..

[16] Fu Y., Zhao X.H. (2013). *In vitro* responses of hFOB1.19 cells towards chum salmon (*Oncorhynchus keta*) skin gelatin hydrolysates in cell proliferation, cycle progression and apoptosis. J. Funct. Foods.

[17] Chen X.X., Deng Y.F., Zhou Z.L., Tao Q.S., Zhu J., Li X.L., Chen J.L., Hou J.F. (2010). 17β-estradiol combined with testosterone promotes chicken osteoblast proliferation and differentiation by accelerating the cell cycle and inhibiting apoptosis *in vitro*. Vet. Res. Commun..

[18] Heath M.C. (1998). Apoptosis, programmed cell death and the hypersensitive response. Eur. J. Plant Pathol..

[19] Bellido T., Plotkin L.I. (2011). Novel actions of bisphosphonates in bone: Preservation of osteoblast and osteocyte viability. Bone.

[20] Ren G., Ferreri M., Wang Z., Su Y., Han B., Su J. (2011). Sodium fluoride affects proliferation and apoptosis through insulin-like growth factor I receptor in primary cultured mouse osteoblasts. Biol. Trace Elem. Res..

[21] Ahuja S.S., Zhao S., Bellido T., Plotkin L.I., Jimenez F., Bonewald L.F. (2003). CD40 ligand blocks apoptosis induced by tumor necrosis factor α, glucocorticoids, and etoposide in osteoblasts and the osteocyte-like cell line murine long bone osteocyte-Y4. Endocrinology.

[22] Pagel C.N., Niese M.R., Abraham L.A., Chinni C., Song S.J., Pike R.N., Mackie E.J. (2003). Inhibition of osteoblast apoptosis by thrombin. Bone.

[23] Grey A., Zhu Q., Watson M., Callon K., Cornish J. (2006). Lactoferrin potently inhibits osteoblast apoptosis, via an LRP1-independent pathway. Mol. Cell. Endocrinol..

[24] Yang R.Y., Zhang Z.F., Pei X.R., Han X.L., Wang J.B., Wang L.L., Long Z., Shen X.Y., Li Y. (2009). Immunomodulatory effects of marine oligopeptide preparation from chum salmon (*Oncorhynchus keta*) in mice. Food Chem..

[25] Martínez-Maqueda D., Miralles B., Recio I., Hernández-Ledesma B. (2012). Antihypertensive peptides from food proteins: A review. Food Funct..

[26] Fu Y., Zhao X.H. (2014). Utilization of chum salmon (*Oncorhynchus keta*) skin gelatin hydrolysates to attenuate hydrogen peroxide-induced oxidative injury in rat hepatocyte BRL cell model. J. Aquat. Food Prod. Technol..

[27] Jiang S.J., Zhao X.H. (2010). Transglutaminase-induced cross-linking and glucosamine conjugation in soybean protein isolates and its impacts on some functional properties of the products. Eur. Food Res. Technol..

[28] Eckert R.L., Katzenellenbogen B.S. (1982). Effect of estrogens and antiestrogen receptor dynamics and the induction of progesterone receptor in MCF-7 human breast cancer cells. Cancer Res..

[29] Kunst T., Whitaker J.R., Voragen A.G.J., Wong D.W.S. (2003). Handbook of Food Enzymology.

[30] Zhou P., Regenstein J.M. (2006). Determination of total protein content in gelatin solutions with the lowry or biuret assay. J. Food Sci..

[31] Church F.C., Swaisgood H.E., Porter D.H., Catignani G.L. (1983). Spectrophotometric assay using *o*-phthaldialdehyde for determination of proteolysis in milk and isolated milk protein. J. Dairy Sci..

[32] Nielsen P.M., Petersen D., Dambmann C. (2001). Improved method for determining food protein degree of hydrolysis. J. Food Sci..

[33] Zhang J., Liu L., Mu X.M., Jiang Z.Z., Zhang L.Y. (2012). Effect of triptolide on estradiol release from cultured rat granulose cells. Endocr. J..

